# Single‐cell profiling reveals various types of interstitial cells in the bladder

**DOI:** 10.1111/cpr.13431

**Published:** 2023-02-23

**Authors:** Jiang Zhao, Chengfei Yang, Bo Liang, Ye Gao, Jing Luo, Ji Zheng, Bo Song, Wenhao Shen, Xingyou Dong, ShuangShuang Dai, Zhenxing Yang

**Affiliations:** ^1^ Department of Urology, Second Affiliated Hospital Army Medical University Chongqing People's Republic of China; ^2^ Department of Biochemistry and Molecular Biology Army Medical University Chongqing People's Republic of China; ^3^ Department of Urology Xiangshan First People's Hospital Medical and Health Group Zhejiang People's Republic of China; ^4^ Department of urology General Hospital of Xinjiang Military Command Xinjiang People's Republic of China; ^5^ Department of Urology, Southwest Hospital Army Medical University Chongqing People's Republic of China; ^6^ Department of Urology People's Hospital of Shapingba District Chongqing People's Republic of China; ^7^ Department of Blood Transfusion, Irradiation Biology Laboratory Army Medical University Chongqing People's Republic of China

## Abstract

Clarifying the locations, molecular markers, functions and roles of bladder interstitial cells is crucial for comprehending the pathophysiology of the bladder. This research utilized human, rat and mouse bladder single‐cell sequencing, bioinformatics analysis and experimental validation. The main cell types found in human, rat and mouse bladder tissues include epithelial cells, smooth muscle cells, endothelial cells, fibroblasts, myofibroblasts, neurons and various immune cells. Our study identified two significant types of interstitial cells (PTN^+^IGFBP6^+^PI16 (CD364)^+^ CD34^+^) and myofibroblasts (STC1^+^PLAT^+^TNC^+^). These two types of interstitial cells are mainly located in the subepithelial lamina propria, between muscles and between muscle bundles. In the CYP (cyclophosphamide)‐induced bladder injury mouse model, the interaction types and signals (MK, MIF, GDF and CXCL) of fibroblasts and myofibroblasts significantly increased compared with the normal group. However, in the aging mouse model, the signals CD34, LAMININ, GALECTIN, MK, SELPLG, ncWNT, HSPG, ICAM and ITGAL‐ITGB2 of fibroblasts and myofibroblasts disappeared, but the signals PTN and SEMA3 significantly increased. Our findings identified two crucial types of interstitial cells in bladder tissue, fibroblasts and myofibroblasts, which play a significant role in normal bladder physiology, CYP‐induced bladder injury and aging bladder development.

## Graphical abstract

Clarifying the locations, molecular markers, functions and roles of bladder interstitial cells is important for understanding the pathophysiology of the bladder. In this paper, the location, molecular markers and functions of bladder interstitial cells were elucidated by sequencing of human, mouse and rat bladder single cells, bioinformatics analysis and experimental verification. In this study, two important types of interstitial cells, fibroblasts (PTN+IGFBP6+PI16 (CD364)+ CD34+) and myofibroblasts (STC1+PLAT+TNC+), were identified. At the same time, we found that two important types of interstitial cells fibroblasts and myofibroblasts could play an important role in normal bladder physiology, CYP‐induced bladder injury and aging bladder development.

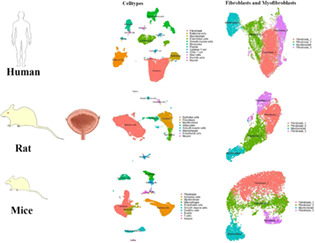

## 1 INTRODUCTION

The bladder's filling and voiding of urine are controlled by a combination of complex neural systems in the spinal cord and peripheral neurons, as well as the muscle cells and urothelium.^1^ Smooth muscle cells are crucial for bladder function, and increased excitation‐contraction coupling in the detrusor muscle results in the voiding of urine from the bladder. The urothelium also acts as a barrier between the blood and urine, preventing reabsorption of harmful compounds from the urine, playing a role in sensory function. This is accomplished through the expression of numerous receptors/ion channels that serve as neuronal sensor molecules.^2^ In contrast, the role of interstitial cells in the bladder is still a subject of debate.^3^ Further research is needed to fully understand the locations, molecular markers, functions and roles of interstitial cells, as well as their contribution to spontaneous contractions of the bladder.

In previous studies, various molecular markers such as c‐Kit, PDGFRα, Desmin, Vimentin, cGMP and CD34 have been utilized to label interstitial cells.^4^, ^5^, ^6^, ^7^ Moreover, the identified bladder interstitial cells were named interstitial cells (ICs), Cajal interstitial cell‐like cells (ICCs), Cajal interstitial cells (ICCs), pacemaker cells and fibroblast‐like cells (FLCs), leading to confusion in the field.^4^, ^5^, ^6^, ^7^, ^8^, ^9^ Recent research has used immunohistochemistry and electron microscopy to identify three types of interstitial cells in the bladder: telocytes, fibroblasts and myofibroblasts.^4^, ^5^, ^6^, ^7^, ^8^, ^9^ Despite these advancements, the exact locations, molecular markers, functions and roles of bladder interstitial cells are yet to be fully understood. The development of single‐cell RNA‐seq (scRNA‐seq) technology, specifically the 10x Genomics platform, has allowed for an unbiased classification of cell types from single‐cell suspensions, and this method has been applied to bladder tissue research with great success.^10^, ^11^, ^12^ Additionally, the utilization of next‐generation sequencing (NGS) enables easy handling and analysis of substantial amounts of RNA‐seq data, leading to improved visualization. This study aims to explore the various cell types present in bladder tissue, with the ultimate goal of gaining new insights into bladder organization and disease occurrences.

## 2 METHODS

### 2.1 Samples

Two normal bladder anterior wall tissues (from one male, 72 years old and one female, 47 years old) were donated by two patients who underwent open radical cystectomy. The current investigation was conducted with the approval of the medical ethics committee of the Second Affiliated Hospital of the Army Medical University. Each patient signed a written informed consent form before donating their tissues for this study. For detailed information about the patients, see the supplementary information.

Two Sprague–Dawley rats (8 weeks, one male and one female) were purchased from the Laboratory Animal Centre of the Third Afflicted Hospital at the Army Medical University. Rats were group‐housed under a 12‐h light/dark cycle and provided with water and food. All experimental procedures were approved by the Army Medical University Animal Care and Use Committee and were performed in accordance with the Institutional Animal Welfare Guidelines.

Five wild‐type C57BL/6J mouse bladder tissues were citied from published paper.^12^ The mouse scRNA‐seq data (detailed information in Table S5) were downloaded from the published database (accession number GSE153562) for analysis. Bladder tissues of newly collected samples were collected from female mouse.

### 2.2 Tissue preparation, handling and enzymatic isolation

Rats were sacrificed by cervical dislocation. Bladder tissues were isolated from the sacrificed rats under aseptic conditions and placed in sterile PBS (phosphate buffered saline) (Gibco, pH 7.4 basic, lot #8119170, China). With sterile forceps and either small sterile scissors or a sterile scalpel, the bladder tissue was minced into small pieces. The minced bladder tissue was transferred to a 50 ml tube for enzymatic digestion.

Donor bladder tissues from humans (size 1 cm × 1 cm × 1 cm) were collected in DMEM‐high glucose medium (Gibco, lot #8119079, China) containing 10% FBS (Gibco, lot #2017490C, Australia) and 5 μg/ml gentamicin (Gibco, lot #2023926, USA). The wells of a sterile 6‐well plate were prefilled with 10 ml of prewarmed tissue washing medium (PBS and 5 μg/ml gentamicin). The tissue was gently agitated in the well using sterile forceps for 5–10 s; successive washing of the tissue was continued through each unused well until all six wells were used. With sterile forceps and either small sterile scissors or a sterile scalpel, the bladder tissue was minced into small pieces. Minced bladder tissue was transferred to a 50 ml tube for subsequent enzymatic digestion. Tissues from rats and humans were digested for approximately 4 h at 37 °C with agitation in a digestion solution containing 2 mg/ml papain (Worthington, lot #LS003120), 100 U/ml Collagenase II (Sigma, CAS No: 9001‐12‐1, USA) and 100 U/ml Collagenase IV (Sigma, CAS No: 9001‐12‐1, USA) in DMEM‐high glucose medium.^11^ The digested suspension was passed through a 60 μm Steriflip (Millipore, CAS No: SCNY00060) and washed twice with washing medium (PBS and 0.04% BSA). Enriched live cells were washed and counted using a haemocytometer with trypan blue. Overall, it took 6–7 h from obtaining the tissues to generating single‐cell suspensions run on the Chromium 10× device.

### 2.3 Library Preparation and Sequencing

After digestion, the single‐cell suspension was used for the quality assessment and counting; the cell survival rate was generally above 80%. Cells were loaded onto the 10× Chromium Single Cell Platform (10× Genomics, https://www.10xgenomics.com/resources/support-documentation/) at a concentration of 1000 cells/μl (Single Cell 3′ library and Gel Bead Kit v.3) following the manufacturer's protocol. The generation of gel beads in emulsion (GEMs), barcoding, GEM‐RT clean‐up, complementary DNA amplification and library construction were all performed according to the manufacturer's protocol. Qubit was used for library quantification before pooling. The final library pool was sequenced on the Illumina HiSeq instrument using 150‐base‐pair paired‐end reads. Briefly, 6–10 billion base calls were generated for each sample. PCR was performed with the same PCR primer cocktail used in TruSeq DNA Sample Preparation. The pipeline of raw data processing and calling was as follows. Quality control of raw data was conducted with Fast QC software (http://www.bioinformatics.babraham.ac.uk/projects/fastqc/). Reads were mapped to reference the genome build using the Burrows–Wheeler Alignment tool (BWA). The duplicate reads were flagged using Picard‐tools (http://picard.sourceforge.net/). Gene expression levels were quantified as transcripts per million (TPM), which were calculated as the number of UMIs of each gene divided by all the UMIs of a given cell and multiplied by 1000,000. Three key parameters (number of RNA features >200 and <7500, percentage of mitochondria <10 and percentage of red cells <10) were used for filtering the raw cells. The parameter of resolution in cluster finding was determined to be 0.6. The function Cell Cycle Scoring in the ‘Seurat’ package with the R platform was used to perform cell division phase analysis based on scRNA‐seq data. The mouse scRNA‐seq data (five female mouse, detailed information in Table S5) were downloaded from the published database (accession number GSE153562) for analysis.

### 2.4 Data processing tools

All data processing was based on the R platform (version 3.6.2, https://www.R-project.org/). Seurat (http://satijalab.org/seurat/install.html) was employed for cell clustering. Pseudotime trajectory analysis was performed with Monocle (http://bioconductor.org/packages/release/bioc/html/monocle.html). Pheatmap (https://cran.r-project.org/web/packages/pheatmap/index.html) was used for plotting the heatmap. Pathway descriptions (GO and KEGG enrichment analysis) were demonstrated by clusterProfiler (http://bioconductor.org/packages/release/bioc/html/clusterProfiler.html). The p values were calculated using Pearson's correlation. Values of *p* < 0.05 were considered significant.

### 2.5 Immunohistochemistry and Immunofluorescence Staining

All antibodies applied in the current assays are listed in Table S6. Briefly, frozen bladder tissue sections were placed directly on slides and then fixed with immunostaining fixative (Beyotime, #P0098, China). Then, the slides were washed twice with immunostaining washing solution (Beyotime, #P0106, China) for 5 min each. The immunostaining blocking solution (Beyotime, #P0102, China) was added and blocked overnight at 4 °C. The primary antibody was diluted to the appropriate ratio according to the instructions with QuickBlock Primary antibody dilution buffer (Beyotime, #P0262, China) and then added to the sections and incubated overnight at 4 °C with slow shaking. Immunostaining washing solution was then added and washed slowly by shaking on a side shaker for 5 min; this was repeated three times. Based on the instruction manual of the secondary antibody, dilution with the secondary antibody buffer for immunofluorescence (Beyotime, #P0106, China) or with secondary antibody solution for immunohistochemistry (Beyotime, #P0110, China) was performed. Immunostaining washing solution was added and washed slowly by shaking on a side shaker for 5 min, which was repeated three times. The results were observed using the appropriate tools.

### 2.6 Flow Cytometric Sorting and Transmission Electron Microscopy (TEM)

The cell surface staining procedure of human cell suspensions was performed according to the instructions of BD Biosciences. Briefly, cells were resuspended in BD Pharmingen Stain Buffer (BSA) (Cat. No. 554657), 1 ml of precooled BSA was added, and the samples were centrifuged at 4 °C for 5 min. This process was repeated two times. The final cell concentration was adjusted to 2 × 10^7^ cells/ml with BSA, and 100 μL of the cell suspension (106 cells) was transferred to a 12 × 75 mm round bottom polypropylene test tube. Then, 5 μL of the surface antibody (PI16) was added to each tube and incubated for 20 min on ice in the dark. The cells were washed twice with an appropriate amount of staining buffer and centrifuged at 300 × *g* for 5 min. The tube or microplate was tapped to mix the cells, and flow cytometry was performed to filter *PI16* ^+^ cells. Cells in a 10 cm dish were collected and fixed with 2.5% glutaraldehyde in phosphate buffer solution for 2 h and rinsed with 0.1 M phosphoric acid for 15 min three times. Subsequently, 1% acetic acid fixative was added for 2–3 h and rinsed with 0.1 M phosphoric acid for 15 min, three times. Dehydration treatment was performed at 4 °C with 50% ethanol for 15 min, 70% ethanol for 15 min, 90% ethanol for 15 min, 90% ethanol and 90% acetone for 15 min, and 90% acetone for 20 min. Cells were embedded in pure embedding solutions at 37 °C for 3 h and cured in a 45 °C oven for 12 h. After ultrathin microtome slicing at 50 nm, the sections were stained with 3% uranium acetate‐lead citrate. The stained sections were observed by TEM, and the results were recorded.

## 3 RESULTS

### 3.1 Identification of cell types in the bladder tissues of humans, rats and mouse

**FIGURE 1:**
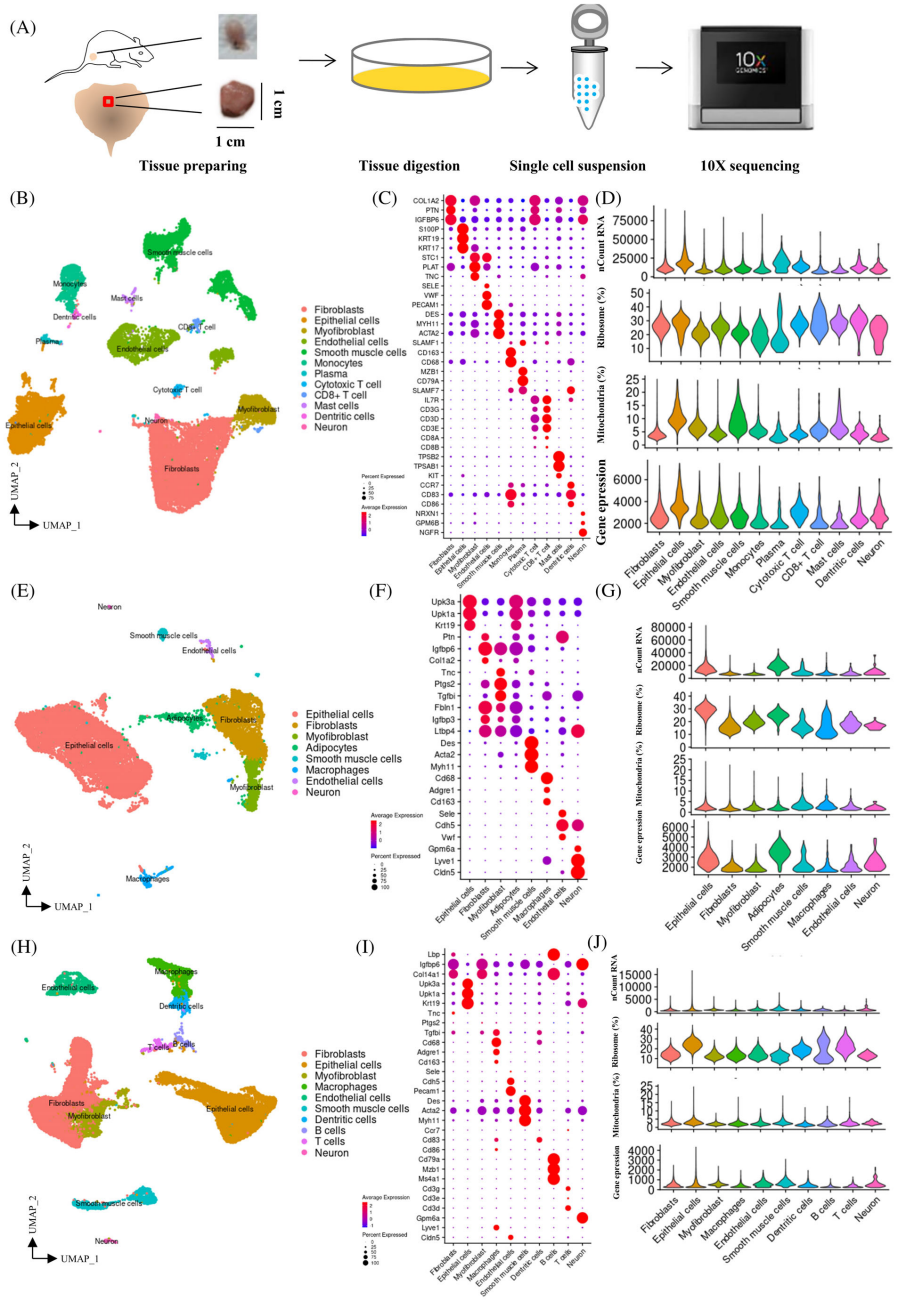
10× Single‐Cell RNA‐seq Analysis of Bladder Tissues. (A) A schematic representation of experimental processing. (B) Uniform manifold approximation and projection (UMAP) analysis of all of the filtered cells (*n* = 27,798) from human bladder tissues. (C) Dot plot showing scaled expression levels for marker genes of each cell type in the human bladder. (D) VlnPlot showing the average expression level of quality control parameters for different cell types in the human bladder. (E) Uniform manifold approximation and projection (UMAP) analysis of all of the filtered cells (*n* = 14,891) from rat bladder tissues. (F) Dot plot showing scaled expression levels for marker genes of each cell type in the rat bladder. (G) VlnPlot showing the average expression level of quality control parameters for different cell types in the rat bladder. (H) Uniform manifold approximation and projection (UMAP) analysis of all of the filtered cells (*n* = 33,622) from mouse bladder tissues. (I) Dot plot showing scaled expression levels for marker genes of each cell type in mouse bladder. (J) VlnPlot showing the average expression level of quality control parameters for different cell types in mouse bladders.

The cells were collected from different zones of the human bladder and from the overall bladder of rats, and were then processed into a single‐cell suspension for scRNA‐seq analysis using the 10x Genomics platform version 3.0 (Figure 1A). scRNA‐seq data from mouse bladders was obtained from the Short Read Archive (accession number GSE153562).^12^ Unsupervised clustering analysis (UMAP, uniform manifold approximation and projection) showed 36, 22 and 28 clusters in human, rat and mouse cells, respectively. Based on bladder histological structure and marker gene characterization (Table S1), 12 major cell clusters were identified in humans, eight in rats and 10 in mice (Figure 1B, E, H). Cells from different sexes were evenly distributed within the same clusters and showed minimal differences (Figure S1A, B, D, E). Common cell types across the three species included epithelial cells, fibroblasts, myofibroblasts, endothelial cells, smooth muscle cells, neurons and macrophages. Differences in cell types mainly lie in immune cells, with mast and plasma cells observed only in human bladders, and adipocytes present only in rat bladders. Three common marker genes were used to distinguish cell types, with minimal differences observed between species (Figure 1C, F, I). The total number of marker genes for different cell types in each species can be found in Tables [Link], [Link]. Quality control data, including nCount RNA, ribosome percentage, mitochondrial percentage and gene expression level, were reasonable across different cell types (Figure 1D, G, J). On average, 2640 genes were detected from human cells, 2496 from rat cells and 1894 from mouse single cells.

### 3.2 Characterization of different cell types in the bladder

**FIGURE 2:**
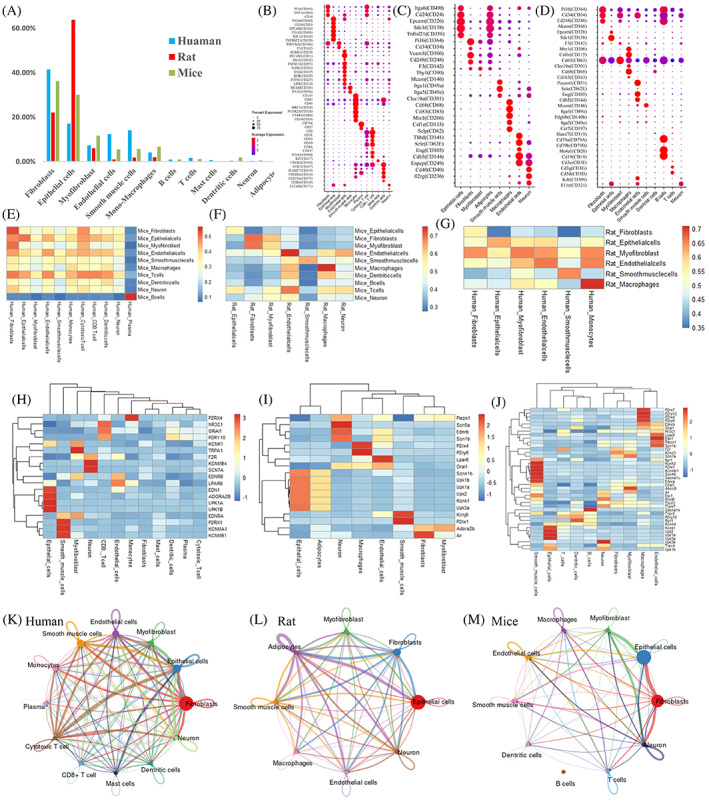
Characterization of different cell types in the bladder. (A) Comparison of the numbers of each cell type in bladder tissues from humans, rats and mouse. Dot plot showing scaled expression levels for CD marker genes of each cell type in human (B), rat (C) and mouse bladder (D). Pearson correlation of the average expression level between cell types from human and mouse (E), rat and mouse (F), and human and rat (G) bladder tissues. Heatmap showing scaled expression levels for receptors and ion channels in different cell types in human (H), rat (I) and mouse (J) bladders. Circle plot denoting cell–cell communication networks among different cell types in human (K), rat (L) and mouse bladder (M).

After strict filtering and quality control (average gene level ≥ 1000), a total of 27,798 cells from two human (one male and one female), 14,891 cells from two rat (one male and one female) and 33,622 cells from five mouse (female, different ages) bladder tissues were obtained for clustering (Figure 2A and Table S5). The number and type of cells showed minimal differences among species (Figure 2A). To further analyse the features of major cell clusters in bladders, we assessed CD markers, ion channels and cell–cell interactions among different cell types. CD molecular markers can specifically label cell populations in bladder tissues, such as *CD49f*, *CD24*, *CD138* and *CD358* for urothelium cells; *CD364* and *CD34* for fibroblasts; *CD142*(*F3*) for myofibroblasts; *CD146* and *CD49a* for smooth muscle cells; *CD31*, *CD105* and *CD62E* for endothelial cells; and other immune‐related cells following the published markers (Figure 2B–D). Pearson correlation analysis showed high consistency between human and rat bladder tissue homologous cell types (Figure 2E–G). One of the main functions of epithelial and smooth muscle cells in the bladder is ion transport and sensory transduction. We also identified epithelial sodium channel Scnn1b in rat, potassium two pore domain channel KCNK1, UPKs protein expressed in human, rat and mouse urothelium cells. Meanwhile, P2RX1, KCNMA1 and KCNMB1 expressed in human smooth muscle cells, TRPA1 expressed in human myofibroblasts, LPAR6 and EDNRB expressed in human, rat and mouse endothelial cells (Figure 2H, I). In addition, to examine cell cycle variation in bladder data, we assigned each cell a score based on its expression of G2/M and S phase markers. The results indicated that the cell division phase (G2M/S) was mainly found in immune cells (Figure S1C, F, I, J). Cell–cell interactions among different cell types play important roles in bladder function. The online platform CellChat was used to predict major signalling inputs and outputs for all cell populations and how these populations and signals coordinate for functions.^13^ In total, extensive signalling interactions exist among fibroblasts, myofibroblasts and epithelial cells in human, rat and mouse bladders (Figure 2K–M). Different signalling pathways were observed in different cell types (Figure S1K–M).

### 3.3 Characterization of fibroblasts and myofibroblasts in the bladder

**FIGURE 3:**
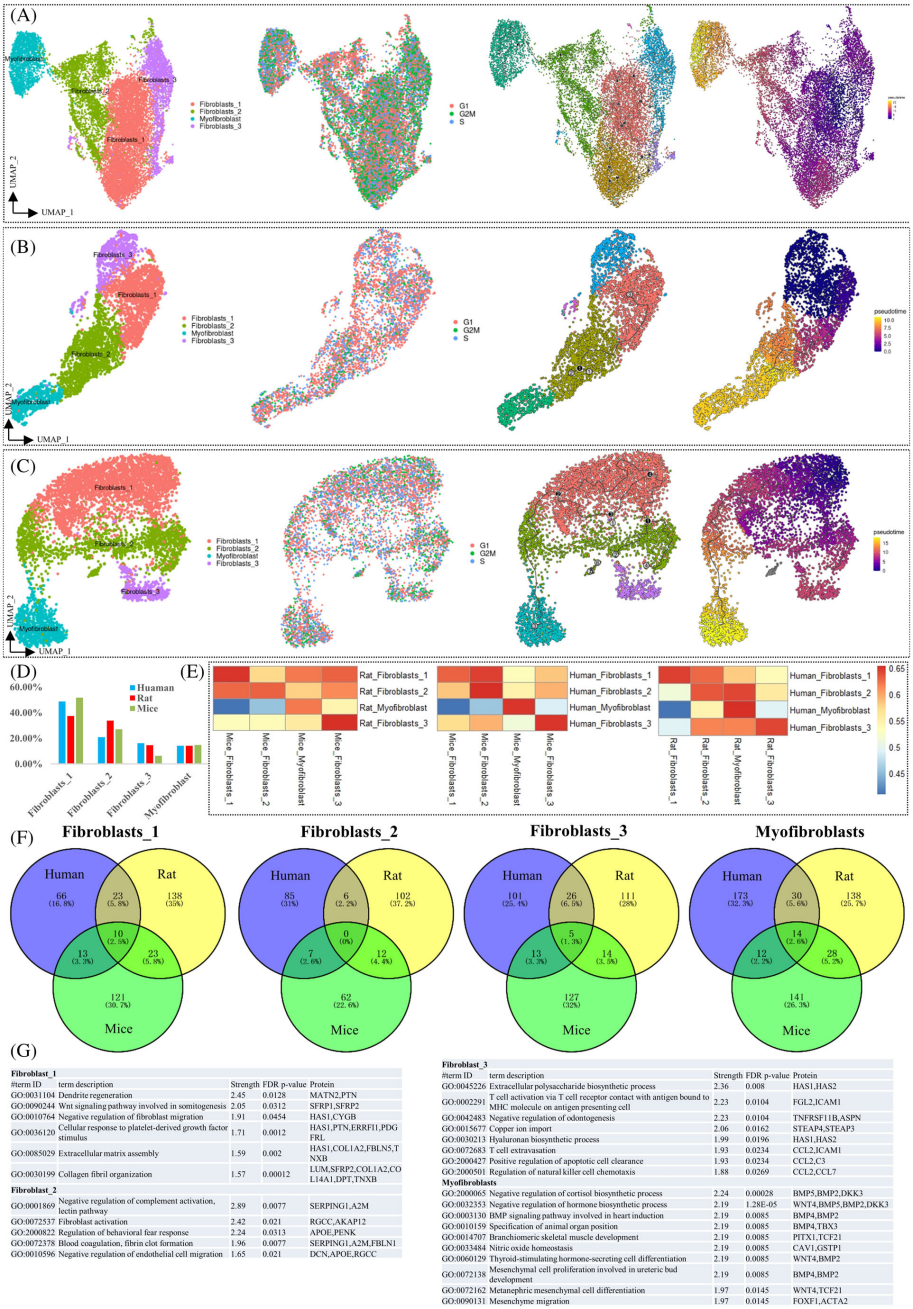
Characterization of fibroblasts and myofibroblasts. (A) UMAP clustering of fibroblast cells (*n* = 11,517), cell cycle phase and pseudotime trajectory analysis from the human bladder. (B) UMAP clustering of fibroblast cells (*n* = 3296), cell cycle phase and pseudotime trajectory analysis from rat bladder. (C) UMAP clustering of fibroblast cells (*n* = 12,157), cell cycle phase and pseudotime trajectory analysis from mouse bladders. (D) Comparison of the numbers of each subpopulation of fibroblasts in bladder tissues from humans, rats and mouse. (E) Pearson correlation analysis of the average expression level between fibroblasts among human, rat and mouse bladder tissue. (F) Venny plot indicating overlapping marker genes among subpopulations in the three species. (G) GO pathway enrichment analysis of highly overlapping marker genes among subpopulations of fibroblasts.

The marker genes for distinguishing fibroblasts were *PTN*, *IGFBP6*, *PI16* (*CD364*) and *CD34* (Figure 2B–D). In addition, second‐level clustering showed three subpopulations of fibroblasts in bladder tissues (Figure 3A–C). Fibroblast types from different sexes and ages were distributed evenly in the same cluster and showed minimal differences (Figure S2A, C, E). No significant cell cycle phase difference exists among three fibroblasts subpopulations (Figure S2G), but pseudotime trajectory analysis demonstrated developmental relationships from fibroblast_1 through fibroblast_2 to fibroblast_3 (Figure 3A–C, Figure S2B, D, F). The number and type of fibroblasts showed minimal differences among the three species (Figure 3D), and Pearson correlation analysis showed high consistency between homologous subpopulations of fibroblasts in human, rat and mouse bladder tissues (Figure 3E). The marker gene expression levels among subpopulations of fibroblasts showed ambiguous boundaries, but overlap marker gene analysis among the three subpopulations indicated different statuses and functions (Figure 3F). Interestingly, GO enrichment analysis of overlapping marker genes in the subgroups of fibroblast_1 identified dendritic regeneration (FDR *p* = 0.0128), the Wnt signalling pathway (FDR *p* = 0.0312), negative regulation of fibroblast migration (FDR *p* = 0.0454) and cellular response to PDGF stimulus (FDR *p* = 0.0012) (Figure 3G). Signalling of fibroblast_2 was related to negative regulation of complement activation (FDR *p* = 0.0077), fibroblast activation (FDR *p* = 0.021) and regulation of behavioural fear response (FDR *p* = 0.0313) (Figure 3G). Signalling of fibroblast_3 was related to the immune response of T‐cell activation (FDR *p* = 0.0104), negative regulation of odontogenesis (FDR *p* = 0.0104) and copper ion import (FDR *p* = 0.0162) (Figure 3G). Thus, fibroblasts can respond to different stimuli, and then activate and participate in the immune response, but its role in bladder signalling transduction still needs further clarification.

**FIGURE 4:**
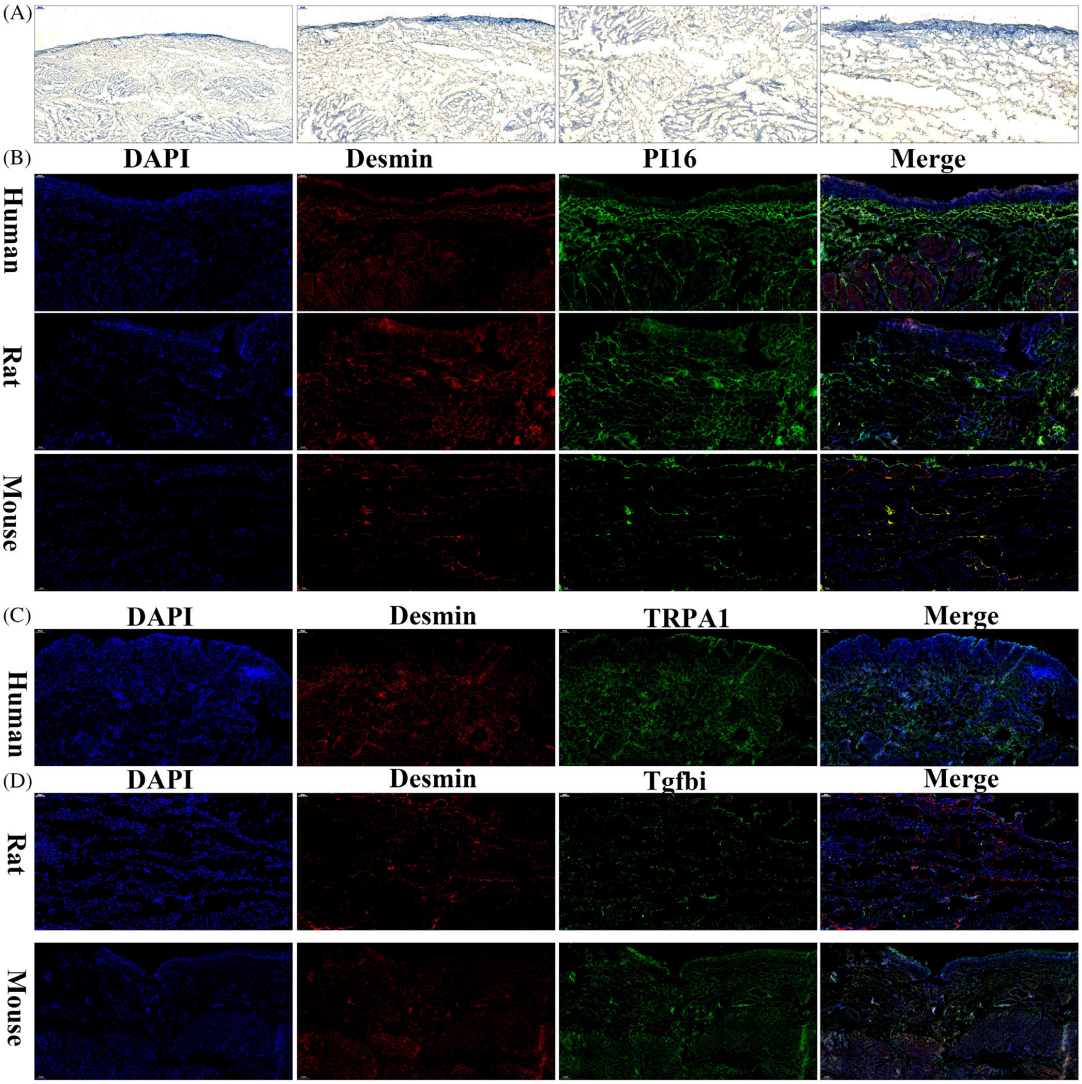
Location of fibroblasts and myofibroblasts in the bladder. (A) Immunohistochemistry staining performed with anti‐PI16 antibody using human bladder tissues. (B) Double immunofluorescence staining performed with anti‐PI16 and anti‐DES antibodies using human, rat and mouse bladder tissues. (C) Double immunofluorescence staining performed with anti‐TRPA1 and anti‐Des antibodies using human bladder tissues. (D) Double immunofluorescence staining performed with anti‐TGFBI and anti‐Des antibodies using rat and mouse bladder tissues.

Fibroblasts, which are one of the key interstitial cells in the bladder, play an important role in bladder tissue and their location must be determined. Through immunohistochemical staining using an anti‐*PI16* antibody, we found that positively stained cells were located in the areas under the epithelial strum, lamina propria and inter muscle bundle (Figure 4A). Flow cytometric sorting of cells from a single cell suspension of human bladder cells using anti‐*PI16* (CD364) antibody showed a fibroblast‐like cell type presentation as a sheet‐like body with prominent cytoplasm, abundance of mitochondria and prominent rough and smooth endoplasmic reticulum (Figure S4). Double immunofluorescence staining performed with anti‐PI16 and anti‐DES (expressed in smooth muscle cells) antibodies on human bladder tissue indicated that fibroblasts are located under the urothelium and among muscular bundle cells (Figure 4B). Similar results were observed in rat and mouse bladder tissues stained with double immunofluorescence, which showed that fibroblasts are in the same location as determined in human bladder tissue (Figure 4B).

The existence of true myofibroblasts in the bladder remains a topic of ongoing discussion. These essentially reactive cells are characterized by the presence of a fibronexus and the expression of marker genes (STC1, PLAT and TNC) found in both muscle cells and fibroblasts (Figure 1C, F, I). Subclustering of fibroblasts revealed that myofibroblasts are distinct from other subtypes (Figures 3A–C), with a high Pearson coefficient of gene expression among the three species (Figure 3D). More interestingly, GO pathway enrichment analysis of overlapping marker genes in myofibroblasts (Figure 3F) identified metanephric mesenchymal cell differentiation and the BMP signalling pathway (BMP genes encode secreted ligands of the TGF‐beta superfamily of proteins). Ligands of this family bind various TGF‐beta receptors, leading to recruitment and activation of SMAD family transcription factors that regulate gene expression. TGF‐beta was the main activation signalling pathway in myofibroblasts and mesenchymal migration (Figure 3G). To locate myofibroblasts in the bladder, we used antibodies against TRPA1 (in this study, we found that TRPA1 specifically expressed in human myofibroblast), *DES* and *TGFBI* were used for double immunofluorescence staining. The results showed that myofibroblasts were located mainly between the urothelium and detrusor (Figure 4C). However, the expression level of *TRPA1* was found to be higher in human myofibroblasts, while it was undetectable in myofibroblasts from rat and mouse tissue (Figures 2H–J). These findings suggest that the function of myofibroblasts in the bladder tissue of humans, rats and mice may still differ and requires further investigation.

### 3.4 The role of fibroblasts and myofibroblasts in CYP (cyclophosphamide)‐induced bladder injury

**FIGURE 5:**
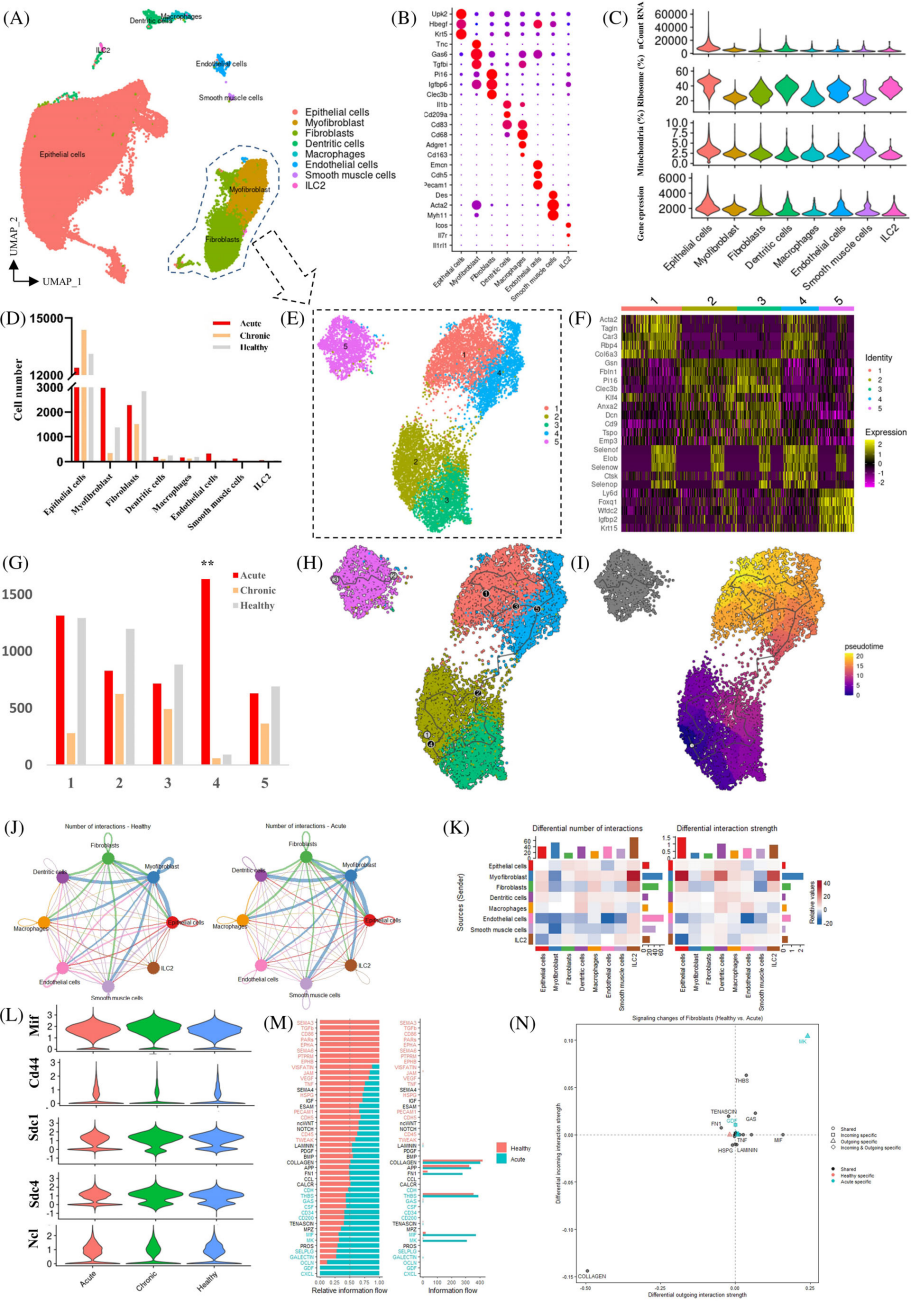
Changes in fibroblasts and myofibroblasts in CYP‐induced bladder injury. (A) UMAP analysis of filtered cells (*n* = 52,912) from mouse bladder tissues. (B) Dot plot showing scaled expression levels for marker genes of each cell type. (C) VlnPlot showing the average expression level of quality control parameters for different cell types in the human bladder. (D) Comparison of the numbers of each cell type in mouse injured bladder tissues. (E) UMAP analysis of fibroblasts and myofibroblasts (*n* = 11,286) from mouse bladder tissues. (F) Heatmap showing the expression of the top five marker genes in different clusters. (G) Comparison of the numbers of each cluster in injured mouse bladder tissues. (H) Pseudotime trajectory analysis of fibroblasts and myofibroblasts in injured mouse bladders. (I) Heatmap showing pseudotime levels for each cluster in mouse bladders. (J) Circle plot of the total number and strength of the inferred cell–cell communication networks in healthy and acute bladder tissues. (K) Number of interactions among different cell populations from healthy and acutely injured bladder tissues. (L) VlnPlot showing the average expression levels of Mif, Cd44, Sdc1, Sdc4 and Ncl in CYP‐treated bladders. (M) Comparison of overall signalling flow within the inferred networks between healthy and acute bladders. (N) Comparison of fibroblast signalling changes between healthy and acutely injured bladders.

To gain a comprehensive understanding of fibroblast and myofibroblast heterogeneity and dynamics in the presence of injury, we analysed published scRNA‐seq data.^14^ In the study, the authors focused on bladder injury as both acute and chronic condition and obtained scRNA‐seq data from the bladder mucosa of adult mice. Although only bladder mucosa tissues were collected and analysed, eight cell types related to the bladder mucosa were identified (Figure 5A). Our data matched the marker gene expression in the study (Figure 5B). No differences were found among the acute, chronic and healthy populations in terms of cell types (Figure S3A). The quality control data was acceptable, with an average of 2133 unique genes detected per cell (Figure 5C). Most cells had three cell cycle phases, but immune cells had prolonged G2M/S periods (Figure S3B, C). After quality control filtering, a total of 18,485 cells from the acute group, 16,502 cells from the chronic group, and 17,925 cells from the healthy group were used for further analysis. The number of epithelial cells was the highest in all three groups, with no significant difference among them (Figure 5D). However, myofibroblasts were mostly present in the acute group, indicating that myofibroblast activation can trigger complex immune responses and control matrix deposition. No significant differences were found among the three groups in terms of the number of cells in other cell types (Figure 5D). Further analysis of fibroblasts and myofibroblasts led to the identification of five clusters in UMAP analysis, with cluster 5 defined as myofibroblasts, independently of other classifications (Figure 5E–F). There were no differences found among the three groups in terms of the subcategories of fibroblasts and myofibroblasts (Figure 5G and Figure S3D). Pseudotime trajectory analysis demonstrated that the developmental relationships of fibroblasts (Figure 5H–I) and myofibroblasts followed their own pattern (Figure S3E, F).

In particular, marker genes in Cluster 5 were selected for GO enrichment analysis, and the results indicated that signalling of endodermal cell differentiation (FDR *p* = 2.96 × 10^−6^), extracellular matrix assembly (FDR *p* = 0.0112) and response to vitamin (FDR *p* = 1.46 × 10^−5^) was activated (Figure S3G, H). Next, we compared the cell–cell interaction signalling between the acute and healthy groups, finding that, overall, the interaction number and strength were higher in bladder tissues with acute injury (Figure 5J), and the number of cell–cell interactions also increased significantly in fibroblasts and myofibroblasts (Figure 5K). Moreover, increased signalling in fibroblasts and myofibroblast cells was unique to acute injury, suggesting the activation of the entire spectrum of communications (Figure 5K). We also compared the relative information flow (the overall communication probability) between the two conditions. Intriguingly, four pathways (MK, MIF, GDF and CXCL) were highly active, albeit at different levels, in acute injury bladder tissues (Figure 5L–N). We found that highly correlated cytokines and receptors, including Mif, Cd44, Sdc1, Sdc4 and Ncl, dominated the signalling patterns (Figure 5L). A closer look at the signalling shows its higher signalling redundancy and high target promiscuity in fibroblasts and myofibroblasts in acute injury bladder tissues (Figure S3I, J).

### 3.5 The role of fibroblasts and myofibroblasts in the aged bladder

**FIGURE 6:**
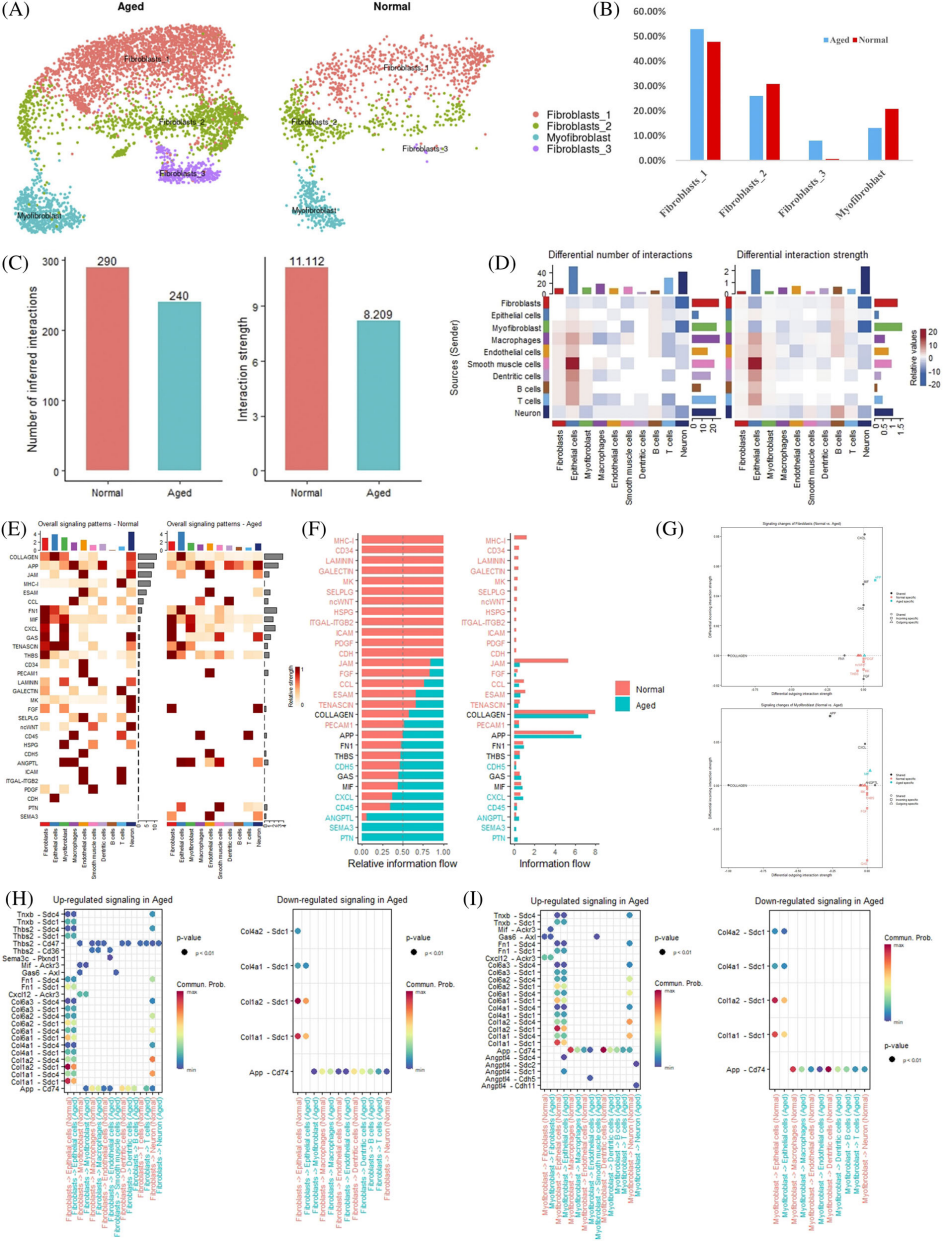
Changes in fibroblasts and myofibroblasts in the aged bladder. (A) UMAP analysis of aged (*n* = 20,924) and normal (*n* = 12,698) group cells from mouse bladder tissues. (B) Comparison of the proportions of each cell type in mouse aged bladder tissues. (C) Comparison of interaction numbers and strength between aged and normal bladder tissues. (D) Heatmap showing interaction numbers and strength in each type of cell between aged and normal bladder tissues. (E) Heatmap showing the overall signalling pattern in each type of cell between aged and normal bladder tissues. (F) Comparison of overall signalling flow within the inferred networks between aged and normal bladder tissues. (G) Comparison of fibroblast signalling changes between healthy and acutely injured bladders. (H) Increased and decreased fibroblast signalling in aged mouse bladders. (I) Increased and decreased myofibroblast signalling in aged mouse bladders.

To better understand fibroblasts and myofibroblasts' change in aging mouse bladder tissues, we divided them in mouse bladder (Figure 3C) into two groups (Figure 6A, normal, age ≤1 years old; aged, age ≥ 18 months). No significant difference in subcategory cell proportions was found between the aged and normal groups (Figure 6B). In terms of cell–cell interaction signalling, aged mouse demonstrated a lower interaction number and strength than normal one (Figure 6C). Additionally, the number and strength of cell–cell interactions decreased in fibroblasts and myofibroblasts (Figure 6D). Analysis of the overall signalling pattern revealed that CD34, LAMININ, GALECTIN, MK, SELPLG, ncWNT, HSPG, ICAM and ITGAL‐ITGB2 signalling disappeared in aged mouse; while PTN and SEMA3 signalling was significantly increased in aged mouse (Figure 6E). We also compared the relative information flow between aged and normal mouse groups, showing the same changes as the overall signalling pattern (Figure 6F). More specifically, signalling changes in fibroblasts included incoming signalling of APP, increased signalling of Thbs2‐Cd47 and decreased signalling of Col4a2/Col4a1/Col1a2/Col1a1‐Sdc1 (Figure 6G, H). Signalling changes in myofibroblasts included outgoing signalling of MIF (Figure 6G), increased signalling of App‐Cd47 and decreased signalling of Col4a2/Col4a1/Col1a2/Col1a1‐Sdc1 (Figure 6I).

## 4 DISCUSSION

In the current study, scRNA‐seq technology was employed to map the transcriptional landscape of human and rat bladder cells. A total of 12, 8 and 10 major cell types were identified in human, rat and mouse bladder tissues respectively, after capturing and filtering cells from the tissues for clustering. In addition to the commonly reported cell types (epithelial cells, smooth muscle cells, endothelial cells and immune cells), we found two types of interstitial cells, that is, fibroblasts and myofibroblasts, which participate in the development of bladder pathology, including drug‐induced cystitis and bladder aging.

Although the major cell clusters did not have a one‐to‐one correspondence across human, rat and mouse tissues, they were coincident with each other. Interstitial cells have been of growing interest in bladder research because they have been linked to the spontaneous contraction activity of the bladder. Interstitial cells have been proven to exist in the bladder of humans and guinea pigs.^7^ Along with immune‐related cells, two types of suburothelial interstitial cells, including fibroblasts and myofibroblasts, were identified.^15^ Buoro et al. first reported the existence of myofibroblasts in the bladder in 1993, and these cells stain for vimentin and α‐smooth muscle actin but not for desmin.^16^ More importantly, the scRNA‐seq data show that myofibroblasts express the specific CD markers *CD362* and *CD121a* and the specific receptor *TRPA1*. Connexin 43 proteins (*GJA1*), cadherin‐11 (*CDH11*), P2Y6 receptors and M3 muscarinic receptors (*CHRM3*) were absent or expressed at low levels in the current research.^17^, ^18^ In summary, myofibroblasts in the submucosal layer of the bladder are linked by gap junctions consisting of JAM3, *GJC1* and *PANX1* with muscle cells and fibroblasts, suggesting a network of functional connections among these cells immediately below the urothelium. Drake et al. reported the morphology, phenotype and ultrastructure of fibroblasts in the human bladder, which was coincident with the current findings.^19^ We found that fibroblasts have the specific markers *PI16* ^+^ *PDGFRα* ^+^/*CD34* ^+^ and the specific CD marker *CD364*(*PI16*). Cultured *PI16* ^+^ fibroblasts displayed ultrastructurally a sheet‐like structure with prominent cytoplasm, abundance of mitochondria, rough or smooth endoplasmic reticulum. They also had a moderately electron dense amorphous content and prominent Golgi complexes. Fibroblastic cells were observed within the lamina propria and throughout the smooth muscle and connective tissue. No specific junction with nerve fibres or smooth muscles was found in fibroblasts. Pathway analysis indicated that fibroblastic cells that respond to different signalling stimuli can interact with smooth muscle cells, epithelial cells and neurons. However, its role in the bladder still needs further validation. Both fibroblasts and myofibroblasts were found in the detrusor layer, and c‐Kit‐expressing cells were confirmed to be mast cells. Although no c c‐Kit^+^ interstitial cells were identified in the study, the existence of such cells in the bladder cannot be ruled out because of the potential technological limitations discussed below. Recent 3D electron microscopic characterization of interstitial cells does not support the incidence of ICC‐like cells in the upper lamina propria of the human bladder.^20^ All in all, whether c‐Kit^+^ interstitial cells exist in the detrusor layer still needs further investigation.

Previous studies have highlighted the role of one cancer‐associated fibroblast subset, named inflammatory cancer‐associated fibroblasts, in bladder cancer carcinoma and have discovered possible therapeutic targets for BC treatment.^21^, ^22^ Studies have shown that fibroblasts participate in the development of cystitis and bladder aging through the signalling of Mif and Sdc. Myofibroblasts have also been reported in a case of an inflammatory myofibroblastic (IMT) tumour of the submandibular gland, which was found in a patient with polyclonal hypergammaglobulinemia and high titers of antinuclear antibody but without any signs of known autoimmune disease.^23^ Additional reports described cases of IMT of the spleen with thrombocytopenic purpura and IMT with Riedel thyroiditis.^24^ Similarly, we found myofibroblast proliferation in CYP‐induced bladder injury involving App‐Cd47 signalling. Research by Matthew et al. showed three subsets of fibroblasts (universal, specialized and disease‐specific) were present in tissues and had a common ancestor source.^25^ Universal and specialized fibroblast subsets coexist in normal, “steady‐state” mouse tissues, and these fibroblasts may be correlated with tissue development. Myofibroblast is the main producer of collagen. Under normal circumstances, collagen connects damaged tissues and becomes a scaffold for wound repair. Su et al. recently revealed the important role of inflammatory factors in the pathogenesis of Interstitial Cystitis/Bladder Pain Syndrome (IC/BPS) through single‐cell transcriptome sequencing.^26^ This study also revealed the “intercellular communication mechanism” of IC/BPS, dominated by fibroblasts. Two inflammatory factors (IL‐6 and TNFα) are key factors in pathogenesis. In the current paper, we found highly correlated cytokines and receptors, including Mif, Cd44, Sdc1, Sdc4 and Ncl dominated signalling patterns contributed to the development of CYP‐induced injury in mouse. However, more mechanistic studies need to be performed in the future.

The current study has some limitations. First, due to difficult process of tissue digestion, some types of cells were captured disproportionately between human, rat and mouse tissues, such as the urothelium cells, fibroblasts and smooth muscle cells. Many connective tissues are present in the bladder, so biases are likely introduced by the different tissue dissociation protocols used.^27^, ^28^ We used a combination of enzymatic dissociation that can avoid biases of the cell populations captured for scRNA‐seq. However, bladder detrusor cells within a bundle are connected to form a functional syncytium, which determines digestion's difficulty. Only a small part of smooth muscle cells was attributed to the detrusor, because a large ratio of captured muscle cells may come from vascular, and even from the muscularis mucosae where smooth muscle fascicles exist freely.^27^, ^29^ Secondly, due to the limitation of cost and sample source, the sample size of this study is small, and only the anterior wall of bladder tissue is captured and digested for analysis; thus, we could not ensure that all cell types were present in the human bladder tissues obtained.

Further, the structure of the bladder neck and urethral sphincter differs from that at the anterior wall, which may lead to the absence of some important cell types related to urination control. However, the whole bladder from rats was analysed for clustering, and we did not find any significant difference between the rat and human samples. Thirdly, only two samples from humans and rats were enrolled for scRNA‐seq analysis, though enough cell numbers were captured using a 10× genomic platform. But current research captured all cell types that emerged in previous studies using scRNA‐seq technology. In addition, in order to exclude interference from other bladders pathological status, two patients who underwent open radical cystectomy and were without bladder disease were enrolled. Fourth, in this study, due to experimental technology and conditions, we failed to isolate and culture myofibroblasts by flow cytometry. Still, we proved the existence of myofibroblasts at the tissue level (Figure S5), and the separation and culture technology of myofibroblasts in the future needs further exploration. Fifth, the existing research techniques and analysis methods have some limitations, resulting in certain limitations of the research results and conclusions.

In conclusion, identifying the cell types in the bladder helps understand the pathophysiology of this organ. This work answered questions regarding the types of bladder interstitial cells, and we identified two types of interstitial cells that participate in the development of bladder pathology, including drug‐induced cystitis and bladder aging.

## AUTHOR CONTRIBUTIONS

Jiang Zhao, Chengfei Yang, ShuangShuang Dai and Zhenxing Yang designed the study; Bo Liang, Ye Gao and Jing Luo collected the data; Ji zheng and Zhenxing Yang performed the bioinformatic analysis. Bo Song and Wenhao Shen drafted the manuscript. Xingyou Dong, Jiang Zhao, Chengfei Yang, ShuangShuang Dai and Zhenxing Yang prepared the figures. All authors read and approved the final version of the manuscript.

## FUNDING INFORMATION

The project was supported partly by grants from National Natural Science Foundation of China (NSFC81873628, 81974101, 81873629).

## CONFLICT OF INTEREST

The authors declare no conflict of interest.

## ETHICS STATEMENT

All experimental procedures were approved by the Army Medical University Animal Care and Use Committee and were performed in accordance with the Institutional Animal Welfare Guidelines.

## Supporting information


**FIGURE S1.** Subclassification of scRNA‐seq data from bladder tissues. (A) UMAP analysis of scRNA‐seq data from male and female bladder tissues of humans. (B) Comparison of the number of each cell type from male and female bladder tissues of humans. (C) Comparison of cell cycle scores of each cell type from human bladder tissues. (D) UMAP analysis of scRNA‐seq data from male and female bladder tissues of rats. (E) Comparison of the number of each cell type from male and female bladder tissues of rats. (F) Comparison of the cell cycle scores of each cell type from rat bladder tissues. (G) UMAP analysis of scRNA‐seq data from bladder tissues of mouse of different ages. (H) Comparison of the number of each cell type from bladder tissues of mouse of different ages. (I) UMAP analysis of scRNA‐seq data from different cell cycle phases. (J) Comparison of the cell cycle scores of each cell type from mouse bladder tissues. (K) Heatmap showing outgoing and incoming signalling patterns in each type of cell from human bladder tissues. (L) Heatmap showing outgoing and incoming signalling patterns in each type of cell from rat bladder tissues. (M) Heatmap showing outgoing and incoming signalling patterns in each type of cell from mouse bladder tissues.

**FIGURE S2.** Subclassification of fibroblasts and myofibroblasts from bladder tissues. (A) UMAP analysis of fibroblast and myofibroblast scRNA‐seq data from male and female bladder tissues of humans. (B) Pseudotime trajectory analysis of fibroblasts and myofibroblasts in different clusters in human bladder tissues. (C) UMAP analysis of fibroblast and myofibroblast scRNA‐seq data from male and female bladder tissues of rats. (D) Pseudotime trajectory analysis of fibroblasts and myofibroblasts in different clusters in rat bladder tissues. (E) UMAP analysis of fibroblast and myofibroblast scRNA‐seq data from bladder tissues of mouse of different ages. (F) Pseudotime trajectory analysis of fibroblasts and myofibroblasts in different clusters in mouse bladder tissues. (G) Comparison of the cell cycle scores of each fibroblast cell type from human, rat and mouse bladder tissues.

**FIGURE S3.** Changes in fibroblasts and myofibroblasts in CYP‐induced bladder injury. (A) UMAP analysis of scRNA‐seq cells from CYP‐induced bladder injury tissues of mouse. (B) UMAP analysis of scRNA‐seq data CYP‐induced bladder injury tissues of mouse with different cell cycle phases. (C) Comparison of the cell cycle scores of each cell type from CYP‐treated bladder tissues. (D) Subcategories of fibroblasts and myofibroblasts from CYP‐induced bladder injury tissues. (E) Heatmap showing pseudotime levels for myofibroblasts in mouse bladders. (F) Pseudotime trajectory analysis of fibroblasts and myofibroblasts in different clusters. (G) Protein–protein interaction analysis of marker genes in Cluster 4. (H) GO enrichment pathway analysis of marker genes in Cluster 4. (I) Increased and decreased fibroblast signalling in CYP‐induced acute bladder injury. (J) Increased and decreased myofibroblast signalling in CYP‐induced acute bladder injury.

**FIGURE S4.** Transmission electron microscope (TEM) scanning of CD364^+^ fibroblast cells from human bladder tissues.

**FIGURE S5.** Transmission electron microscope (TEM) scanning of myofibroblast cells from human bladder tissues.

**TABLE S1.** Marker genes from the literature

**TABLE S2.** Expression of marker gene in human bladder

**TABLE S3.** Expression of marker gene in rat bladder

**TABLE S4.** Expression of marker gene in mice bladder

**TABLE S5.** Patient and mousemanifest ‐ Clinical features of the data.

**TABLE S6.** Detail Antibodies information used in current investigation

## Data Availability

All relevant data are available from the authors. Single‐cell RNA‐seq is available in the Short Read Archive under accession number GSE164557.
