# Supplementary figures and images for: Single‐cell profiling reveals various types of interstitial cells in the bladder
Source: Cell Prolif. 2023 Feb 23;56(9):e13431. doi: 10.1111/cpr.13431 (PMC10472517; doi:10.1111/cpr.13431)

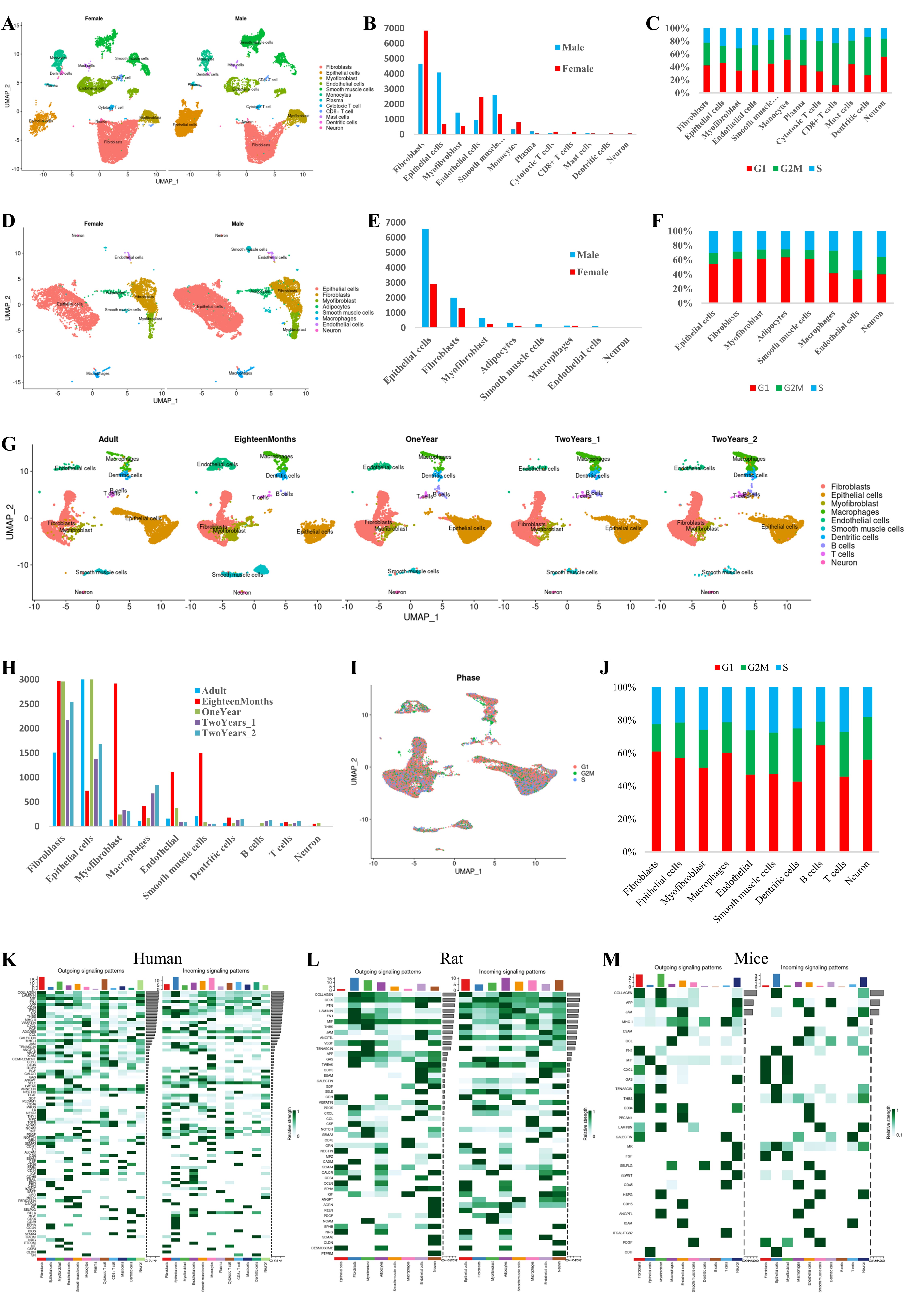

Supplement: Supplementary file 1 — FIGURE S1. Subclassification of scRNA‐seq data from bladder tissues. (A) UMAP analysis of scRNA‐seq data from male and female bladder tissues of humans. (B) Comparison of the number of each cell type from male and female bladder tissues of humans. (C) Comparison of cell cycle scores of each cell type from human bladder tissues. (D) UMAP analysis of scRNA‐seq data from male and female bladder tissues of rats. (E) Comparison of the number of each cell type from male and female bladder tissues of rats. (F) Comparison of the cell cycle scores of each cell type from rat bladder tissues. (G) UMAP analysis of scRNA‐seq data from bladder tissues of mouse of different ages. (H) Comparison of the number of each cell type from bladder tissues of mouse of different ages. (I) UMAP analysis of scRNA‐seq data from different cell cycle phases. (J) Comparison of the cell cycle scores of each cell type from mouse bladder tissues. (K) Heatmap showing outgoing and incoming signalling patterns in each type of cell from human bladder tissues. (L) Heatmap showing outgoing and incoming signalling patterns in each type of cell from rat bladder tissues. (M) Heatmap showing outgoing and incoming signalling patterns in each type of cell from mouse bladder tissues. [file CPR-56-e13431-s005.jpg]

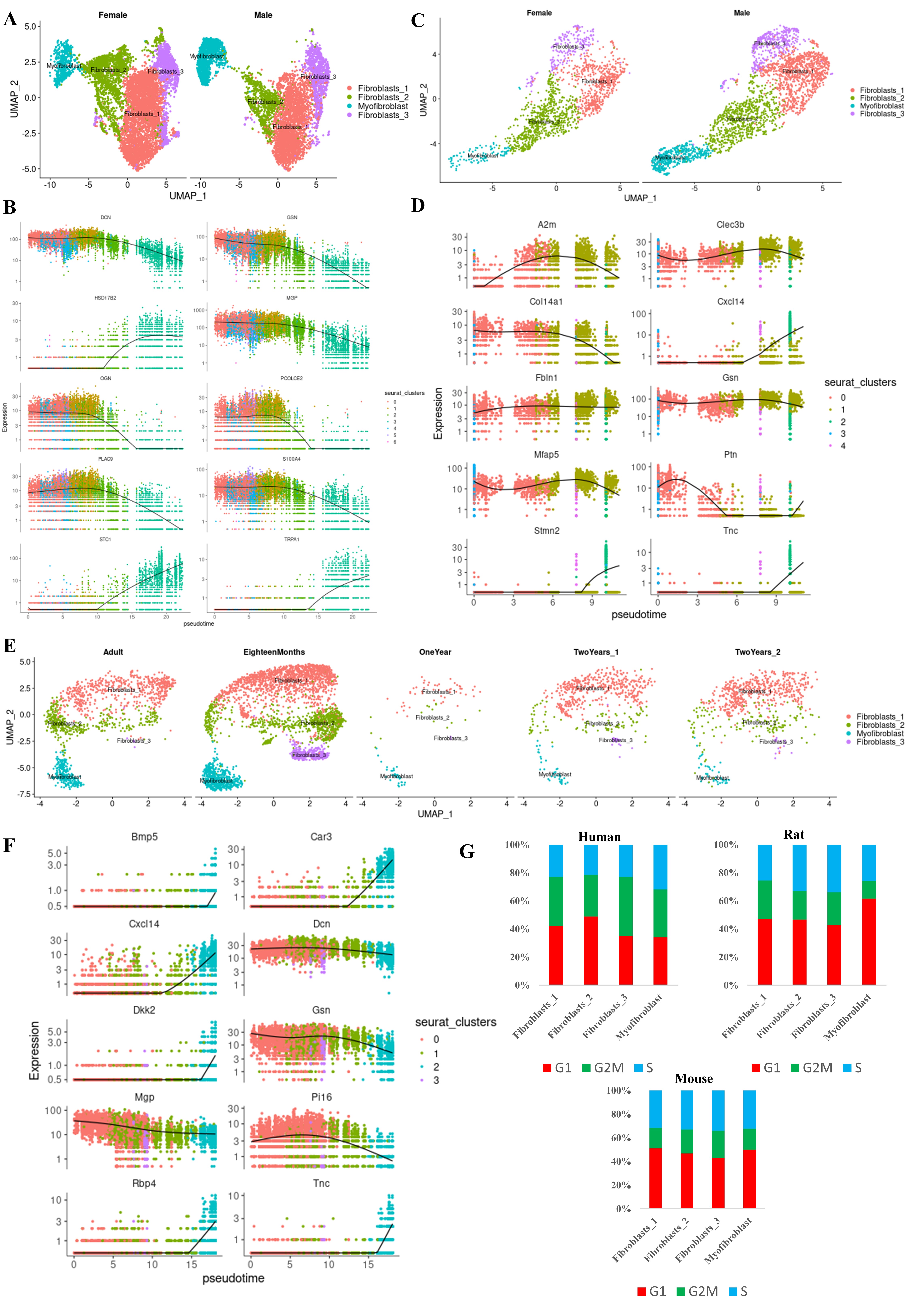

Supplement: Supplementary file 2 — FIGURE S2. Subclassification of fibroblasts and myofibroblasts from bladder tissues. (A) UMAP analysis of fibroblast and myofibroblast scRNA‐seq data from male and female bladder tissues of humans. (B) Pseudotime trajectory analysis of fibroblasts and myofibroblasts in different clusters in human bladder tissues. (C) UMAP analysis of fibroblast and myofibroblast scRNA‐seq data from male and female bladder tissues of rats. (D) Pseudotime trajectory analysis of fibroblasts and myofibroblasts in different clusters in rat bladder tissues. (E) UMAP analysis of fibroblast and myofibroblast scRNA‐seq data from bladder tissues of mouse of different ages. (F) Pseudotime trajectory analysis of fibroblasts and myofibroblasts in different clusters in mouse bladder tissues. (G) Comparison of the cell cycle scores of each fibroblast cell type from human, rat and mouse bladder tissues. [file CPR-56-e13431-s004.jpg]

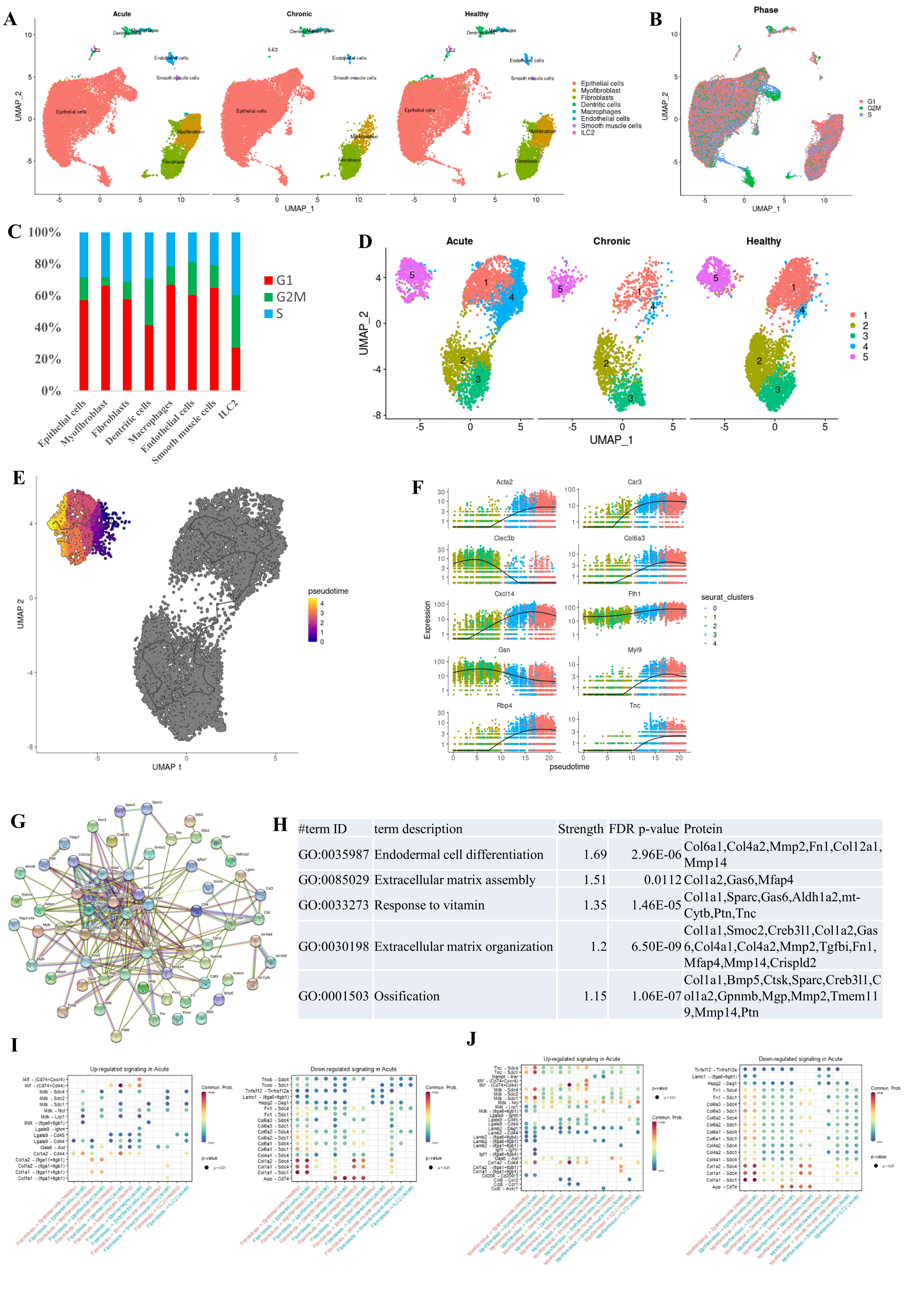

Supplement: Supplementary file 3 — FIGURE S3. Changes in fibroblasts and myofibroblasts in CYP‐induced bladder injury. (A) UMAP analysis of scRNA‐seq cells from CYP‐induced bladder injury tissues of mouse. (B) UMAP analysis of scRNA‐seq data CYP‐induced bladder injury tissues of mouse with different cell cycle phases. (C) Comparison of the cell cycle scores of each cell type from CYP‐treated bladder tissues. (D) Subcategories of fibroblasts and myofibroblasts from CYP‐induced bladder injury tissues. (E) Heatmap showing pseudotime levels for myofibroblasts in mouse bladders. (F) Pseudotime trajectory analysis of fibroblasts and myofibroblasts in different clusters. (G) Protein–protein interaction analysis of marker genes in Cluster 4. (H) GO enrichment pathway analysis of marker genes in Cluster 4. (I) Increased and decreased fibroblast signalling in CYP‐induced acute bladder injury. (J) Increased and decreased myofibroblast signalling in CYP‐induced acute bladder injury. [file CPR-56-e13431-s001.jpg]

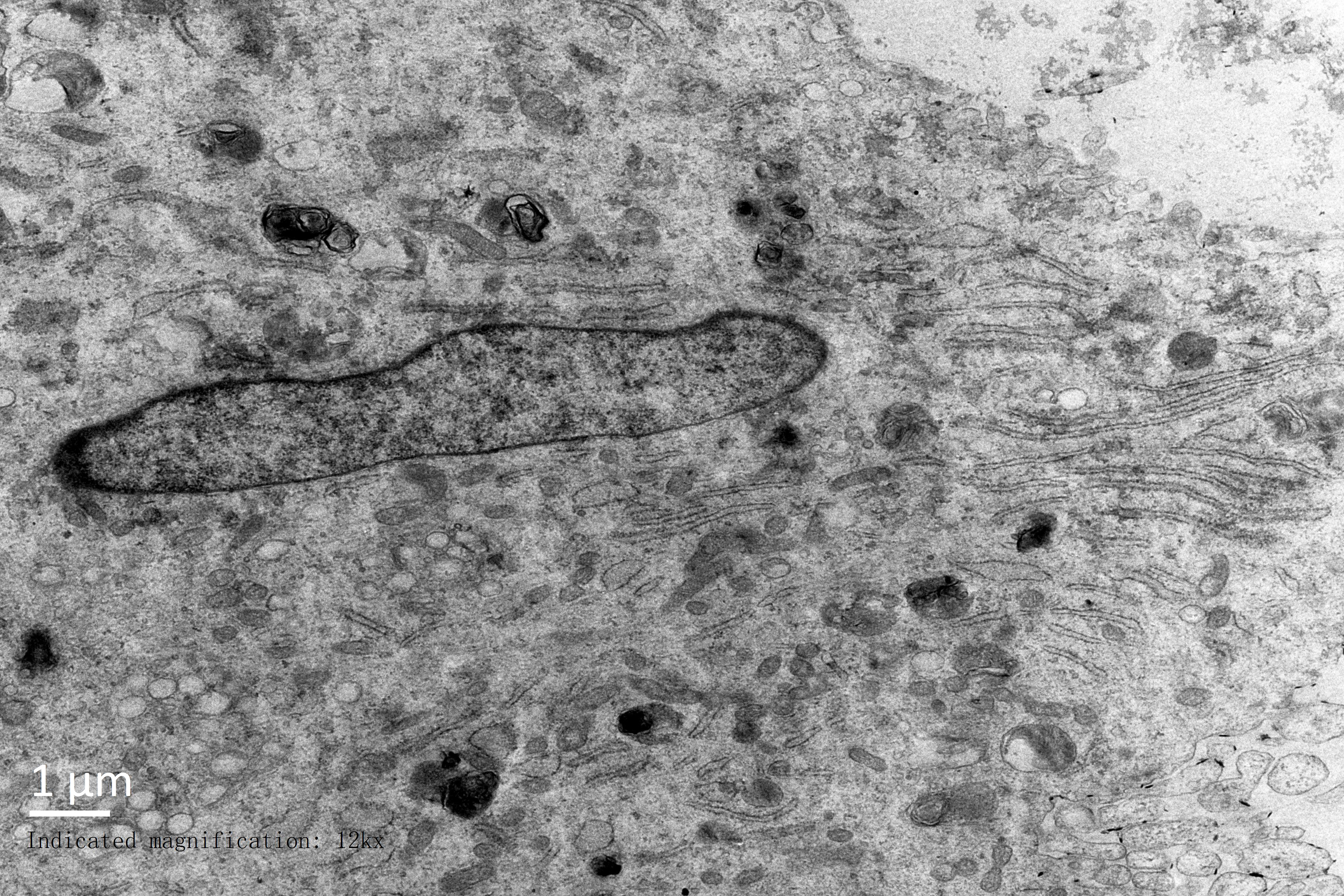

Supplement: Supplementary file 4 — FIGURE S4. Transmission electron microscope (TEM) scanning of CD364+ fibroblast cells from human bladder tissues. [file CPR-56-e13431-s011.jpg]

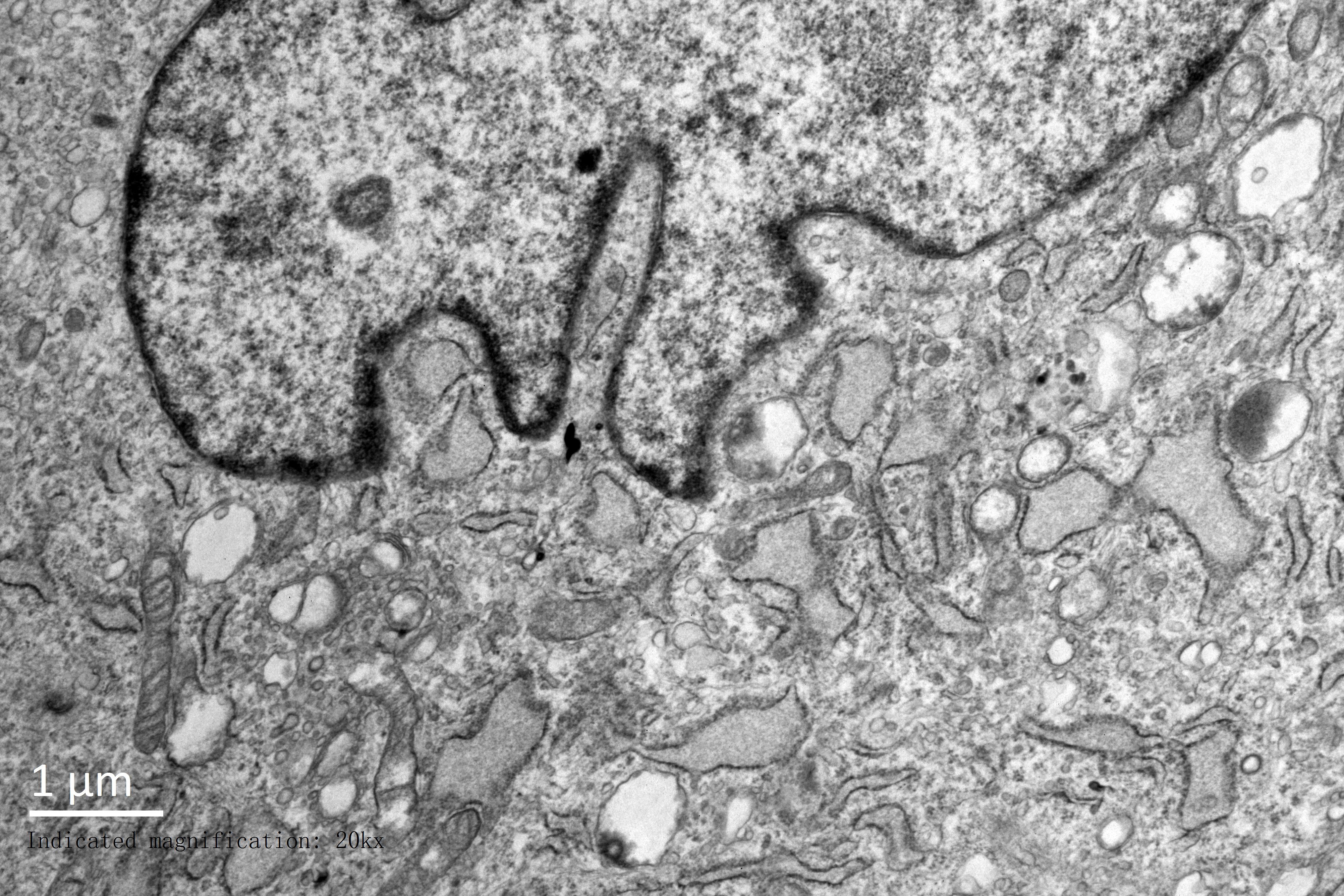

Supplement: Supplementary file 5 — FIGURE S5. Transmission electron microscope (TEM) scanning of myofibroblast cells from human bladder tissues. [file CPR-56-e13431-s003.jpg]
